# Enhancing Vanadium Redox Flow Battery Performance with ZIF-67-Derived Cobalt-Based Electrode Materials

**DOI:** 10.3390/molecules29215061

**Published:** 2024-10-26

**Authors:** Christine Young, Zhen-Qi Liao, Dong-Rong Li, Pei-Ling Li, Chen-Yang Wang, Shu-Mei Ho, Chi-Chang Chen

**Affiliations:** 1 Department of Chemical and Materials Engineering, National Yunlin University of Science and Technology, Yunlin 640301, Taiwan; 2 Green Energy and Environment Research Laboratories, Industrial Technology Research Institute, Tainan 711010, Taiwan

**Keywords:** vanadium redox flow battery, ZIF-67, electrochemical evaluation, energy efficiency

## Abstract

Vanadium redox flow batteries (VRFBs) have emerged as a promising energy storage solution for stabilizing power grids integrated with renewable energy sources. In this study, we synthesized and evaluated a series of zeolitic imidazolate framework-67 (ZIF-67) derivatives as electrode materials for VRFBs, aiming to enhance electrochemical performance. Four materials—Co/NC-700, Co/NC-800, Co_3_O_4_-350, and Co_3_O_4_-450—were prepared through thermal decomposition under different conditions and coated onto graphite felt (GF) electrodes. X-ray diffraction (XRD), scanning electron microscopy (SEM), and transmission electron microscopy (TEM) analyses confirmed the structural integrity and distribution of the active materials. Electrochemical evaluations revealed that electrodes with ZIF-67-derived coatings exhibited significantly lower charge transfer resistance (R_ct_) and higher energy efficiency (EE) compared to uncoated GF electrodes. Co/NC-800//GF delivered the highest energy efficiency and discharge capacity among the tested configurations, maintaining stable performance over 100 charge–discharge cycles. These results indicate that Co/NC-800 holds great potential for use in VRFBs due to its superior electrochemical activity, stability, and scalability.

## 1. Introduction

As the issue of fossil fuel shortages becomes more pressing, renewable energy has become the primary focus of development for many countries. However, renewable energy sources like solar and wind power face challenges due to their intermittent output, which significantly impacts the stability of a power grid. To address this shortcoming, energy storage devices are used to store excess electricity generated during peak production times and release it during periods of low production. This complementary approach can enhance the stability of the power grid when integrated with renewable energy sources [1]. To date, various types of energy storage devices have been developed for different applications, each with its own advantages and limitations. Depending on the applicable use, performance requirements, and costs, the most ideal storage methods can be identified. Energy storage methods can be categorized into several types, with physical storage (such as compressed air energy storage and pumped hydro storage) and chemical storage (such as secondary batteries) being the most widely discussed technologies [2]. Compressed air energy storage involves compressing air into a specific underground space for storage and releasing it to a thermal power turbine when electricity is needed to generate power. However, this method is limited by geological conditions, requiring large air reservoirs or airtight caverns, making it less common in practical applications. Pumped hydro storage utilizes surplus electricity to pump water from a lower reservoir (downstream) to an upper reservoir (upstream). During peak electricity demand, the energy difference between the upper and lower reservoirs is used to generate power. Pumped hydro storage is the most common energy storage system globally, but the construction of reservoirs requires specific geographic conditions and large bodies of water. Additionally, building reservoirs could alter or damage regional ecosystems, limiting the ability to construct them based on storage needs. Secondary batteries, which can be recharged after their energy is depleted, do not have geographic limitations, allowing them to be installed anywhere. In addition, they can provide substantial power and capacity and can charge and discharge within a wide operational window, with a relatively minor environmental impact. Common energy storage batteries include lead–acid batteries, nickel–metal hydride batteries, nickel–cadmium batteries, and lithium–ion batteries [3].

In large-scale energy storage systems, flow batteries (FBs) are a type of secondary battery that operates based on redox principles for charging and discharging. They offer advantages such as long cycle life, high safety, and flexible power and energy scalability [4]. This scalability allows them to be integrated with renewable energy systems, such as photovoltaic solar power and wind farms. FBs typically use aqueous electrolytes, which provide higher safety and eliminate the risk of explosion. These batteries store chemical energy by utilizing different active materials in various oxidation states. Depending on the type of active material and its electrochemical potential, many types of FBs have been developed, including Fe/Cr flow batteries [5], vanadium redox flow batteries (VRFBs) [6,7], Zn/Br_2_ flow batteries [8], and polysulfide/Br_2_ flow batteries [9].

VRFBs are among the most promising and widely researched flow battery technologies, particularly for large-scale energy storage applications. The key redox reactions involved are V^4+^/V^5+^ and V^2+^/V^3+^, with sulfuric acid serving as the solvent in the positive and negative half-cells. The electrochemical charge–discharge reactions for a VRFB can be represented by the following equations:

At the positive electrode (cathode): ${VO}^{2+}+H_{2}O⇌{{VO}_{2}}^{+}+{2H}^{+}+e^{-}$

At the negative electrode (anode): $V^{3+}+e^{-}⇌V^{2+}$

Overall reaction: ${VO}^{2+}+H_{2}O+V^{3+}⇌{{VO}_{2}}^{+}+V^{2+}+{2H}^{+}$

VRFBs offer several advantages, including long cycle life, high safety, scalability, efficiency, stable performance, wide temperature tolerance, and low environmental impact [10]. These features make VRFBs an excellent candidate for grid energy storage, renewable energy integration, and other large-scale applications requiring reliability and longevity. A typical VRFB cell assembly includes components such as an end plate, current collector, bipolar plate, electrode gasket, and ion exchange membrane. The electrolyte, which consists of vanadium in different oxidation states, is stored in two separate tanks to prevent degradation and spontaneous electricity generation. The vanadium–sulfuric acid electrolyte has a long lifespan, demonstrating excellent charge–discharge cycle performance with minimal degradation over time.

There are many factors that influence the charge–discharge efficiency of VRFBs, and one key factor is the electrode material. Since the electrolyte in VRFBs is acidic, the electrode material used in these batteries must have properties such as corrosion resistance, high conductivity, high mechanical strength, and high electrochemical activity. Among various electrode materials, carbon-based materials are widely used in vanadium flow batteries. Common carbon materials include graphite felt (GF), carbon paper, and glassy carbon. However, the performance of VRBs can be affected by the relatively low electrochemical activity and hydrophilicity of these carbon materials. To address these limitations, various modification techniques for carbon materials have been developed. These techniques include thermal activation, acid treatment activation, potassium hydroxide activation, nitrogen doping, the addition of conductive agents, and the use of catalysts [11,12,13]. The primary goals of these methods are to enhance material performance by increasing the oxygen-containing functional groups and hydrophilicity, expanding the active surface area for redox reactions, improving conductivity, and boosting electrochemical activity.

The electrode materials used in vanadium redox flow batteries (VRBs) are primarily divided into two categories: metal and carbon-based materials. Noble metal electrode materials include Au, Pt, Ir, and Pd [14]. However, the high cost of these metals hinders their widespread industrial adoption. Thus, developing advanced metal catalysts for GF that are both cost-effective and can be easily modified remains a challenging task. Introducing transition metal or metal oxide particles onto GF fibers has been shown to not only improve conductivity and catalytic activity but also inhibit hydrogen evolution [15]. For example, He et al. introduced a flexible carbon nanofiber embedded with TiO_2_ (CNF/TiO_2_) prepared via electrospinning for use as a negative electrode in VRFBs [16]. The CNF/TiO_2_ composite demonstrated significantly improved electrochemical performance and energy efficiency compared to pristine CNF electrodes. Yun et al. fabricated NiO-decorated GF electrodes by thermal decomposition, which enhances electrocatalytic activity, improves mass transfer, and significantly boosts VRFB performance, achieving higher energy efficiency and stability over extended cycling [17]. Wang et al. presented a gradient-distributed NiCo_2_O_4_ nanorod-composed GF electrode, designed to address nonuniform mass transport issues in flow batteries. The electrode showed significantly reduced local current density variation, resulting in high energy efficiency (88.81% at 100 mA cm^−2^), superior operational current density (500 mA cm^−2^), and long-term stability (162 h, 200 cycles) [18]. Jiang et al. reported a binder-free MnO_2_ nanosheet array-decorated carbon paper as a negative electrode for VRFBs [19]. The electrochemical performance was optimized using specific solution conditions and deposition time and demonstrated significantly improved electrocatalytic activity for the V^3+^/V^2+^ redox reaction, resulting in higher discharge capacity, better capacity retention, and enhanced energy efficiency. Although these materials demonstrated good performance, their fabrication methods, such as in situ growth of active materials and thermal decomposition, can damage the carbon fiber, potentially affecting its mechanical strength and reducing its lifespan. Additionally, the in situ growth method is challenging to control and difficult to scale up for large-scale production. Ensuring uniform distribution of active materials on the carbon cloth also remains a concern.

In this study, we employed zeolitic imidazolate framework-67 (ZIF-67), a type of metal-organic framework (MOF), as a sacrificial template to fabricate its derivatives for use as electrode materials in VRFBs. MOFs like ZIF-67 are attractive for this purpose due to their large surface area, uniform pore structures, and the presence of metal ions and organic ligands, which make them ideal candidates for synthesizing metal oxides and nanoporous carbon. ZIF-67, which contains cobalt, can be thermally decomposed into cobalt compounds and nanoporous carbon, retaining the original morphology and porous structure of the MOF. Given the remarkable performance of cobalt oxides as electrode materials in VRFBs, we developed a series of ZIF-67-derived materials [20,21,22]. These materials were coated onto graphite felts and assembled into electrodes, incorporating essential components such as end plates, current collectors, bipolar plates, electrode gaskets, and ion exchange membranes to construct a full VRFB. The full flow cell operation was then evaluated through charge–discharge analysis.

## 2. Experimental

### 2.1. Materials

The 2-Methylimidazole (98%) was purchased from Thermo Scientific (Waltham, MA, USA), and Co(NO_3_)_2_·6H_2_O (98 wt%) was obtained from Alfa Aesar (Haverhill, MA, USA). Methanol (98%) was sourced from Reagents Duksan (Ansan, Republic of Korea), and hydrofluoric acid (HF, >48%) from Honeywell Fluka (Seelze, Germany). VOSO_4_ was obtained from Plum-Monix Industry Co., Ltd. (Taoyuan, Taiwan). Graphite felt was obtained from CeTech Co., Ltd. (Taichung, Taiwan). All of these chemicals were used without further purification.

### 2.2. Synthesis

Graphite felt (GF) was initially subjected to thermal treatment at 500 °C for 5 h under an air flow of 1 L/min.

Weigh 0.984 g of 2-methylimidazole and 0.873 g of Co(NO_3_)_2_·6H_2_O, and dissolve them in 40 mL of methanol. Stir the solution for 20 min, then let it stand at room temperature for several hours. After centrifugation and drying overnight, ZIF-67 is obtained. The ZIF-67 is then subjected to further processing. First, it is annealed at 700 °C and 800 °C for 2 h each, with a ramp rate of 5 °C/min under a nitrogen atmosphere. The resultant black powder is then washed with a 10 wt% HF aqueous solution, yielding products denoted as Co/NC-700 and Co/NC-800. Additionally, ZIF-67 is treated at 450 °C for 2 h with a ramp rate of 5 °C/min under a nitrogen atmosphere. The resultant product is then annealed at 350 °C for 1.5 h with a ramp rate of 5 °C/min in air, producing a product denoted as Co_3_O_4_-450. For comparison, ZIF-67 is directly annealed at 350 °C for 2 h with a heating rate of 5 °C/min, resulting in a product denoted as Co_3_O_4_-350.

### 2.3. Characterization

The morphologies of the synthesized samples were characterized using scanning electron microscopy (SEM) and transmission electron microscopy (TEM). SEM images were captured with a JEOL JSM-6701F (JEOL Ltd., Akishima, Japan), while TEM analysis was carried out using a JEOL JEM-2100Plus microscope (JEOL Ltd., Akishima, Japan). X-ray diffraction (XRD) patterns were recorded using a Rigaku MiniFlex-600 (Rigaku Corporation, Akishima, Japan), equipped with Cu Kα radiation. Raman spectroscopy was performed with a Renishaw Invia Raman microscope (Renishaw, Wotton-under-Edge, UK), utilizing a 633 nm laser. X-ray photoelectron spectroscopy (XPS) was conducted using a PHI Versa Probe 4 (Physical Electronics, Chanhassen, MN, USA), employing Al Kα radiation.

### 2.4. Electrochemical Measurements

Add 25 mg of the active material (Co/NC-700, Co/NC-800, Co_3_O_4_-450, and Co_3_O_4_-350) to 500 µL of IPA, then add 250 µL of DI water, and finally mix in 125 µL of 5% Nafion solution to form a slurry. Stir the mixture continuously for 2–3 h using a shaker to ensure no particles settle at the bottom before proceeding with the coating process. For coating, use a fine watercolor brush to gently apply the slurry on the graphite felt (GF). After each coating, place each GF in an oven to dry, and continue the process until the slurry is used up. The mass loading is around 1.3 mg cm^−2^. The coated GF was employed as the positive, and the pristine GF was used as the negative electrode, which is denoted as Co/NC-700//GF, Co/NC-800//GF, Co_3_O_4_-450//GF, and Co_3_O_4_-350//GF. For comparison, GF//GF were also employed under the same condition.

**Figure 1:**
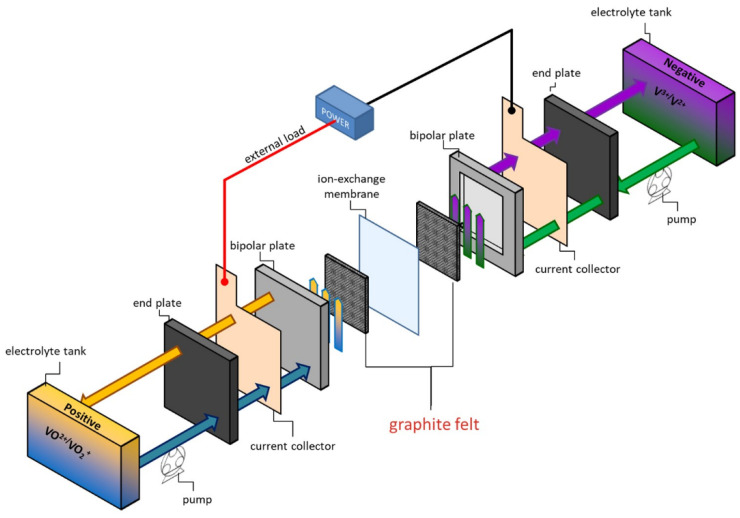
The schematic diagram of a full VRFB cell.

A charge–discharge test was performed in a full VRFB cell (Figure 1) by using a battery testing system (Autolab/PGSTAT302N, Metrohm Autolab, Utrecht, The Netherlands). A 50 mm × 50 mm GF electrode was employed in each bipolar plate. An ion exchange membrane (Nafion^TM^ 212, Chemours, Wilmington, DE, USA) was utilized as a separator. The copper plate was used as a current collector. The cell was connected to two electrolyte reservoirs containing 30 mL of 1.6 M VOSO_4_ in 3 M H_2_SO_4_ for the positive side and for the negative side. The flow rate of electrolyte was maintained at 70 mL min^−1^. A charge–discharge test was carried out at a constant current density of 50, 100, 150, 200, and 250 mA cm^−2^. The EIS test was conducted with the frequency range from 10^−3^ Hz to 10^6^ Hz. Coulombic efficiency (CE), voltage efficiency (VE), and energy efficiency (EE) of the VRFB were calculated as follows:

(1) $$CE=\frac{Q_{discharge}}{Q_{charge}}\times 100\%$$

(2) $$EE=\frac{\int V_{discharge}I_{discharge}}{\int V_{charge}I_{charge}}\times 100\%$$

(3) $$VE=\frac{EE}{CE}\times 100\%$$

where *Q_discharge_* and *Q_charge_* represent the discharge and charge capacity. *V_discharge_* and *V_charge_* are the instantaneous discharge and charge voltage at the currents *I_discharge_* and *I_charge_*, respectively.

## 3. Results and Discussion

**Figure 2:**
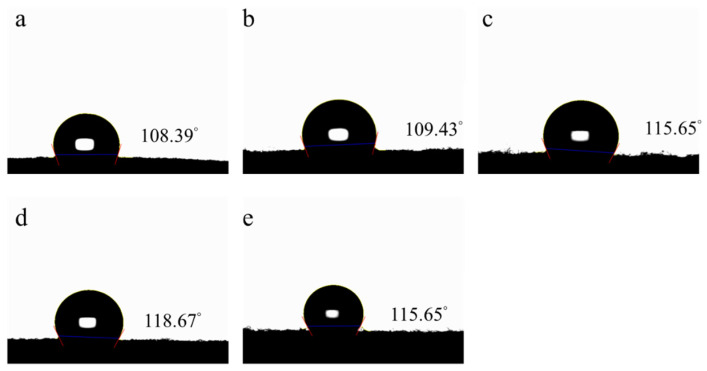
The contact angles of (**a**) graphite felt, (**b**) Co/NC-700, (**c**) Co/NC-800, (**d**) Co_3_O_4_-450, and (**e**) Co_3_O_4_-350.

Co/NC-700, Co/NC-800, Co_3_O_4_-450, and Co_3_O_4_-350 were prepared by using ZIF-67 as the templates and then calcined in different thermal conditions. To assess the wettability of the graphite felt samples, water contact angle measurements were taken by observing the angle formed by a spherical water droplet on the surface (Figure 2). The uncoated graphite felt exhibited a hydrophobic nature, with a water contact angle of 108.39°. After coating with Co/NC-700, Co/NC-800, Co_3_O_4_-450, and Co_3_O_4_-350, the contact angles were found to be 109.43°, 115.65°, 118.67°, and 115.65°, respectively, all indicating hydrophobic characteristics.

**Figure 3:**
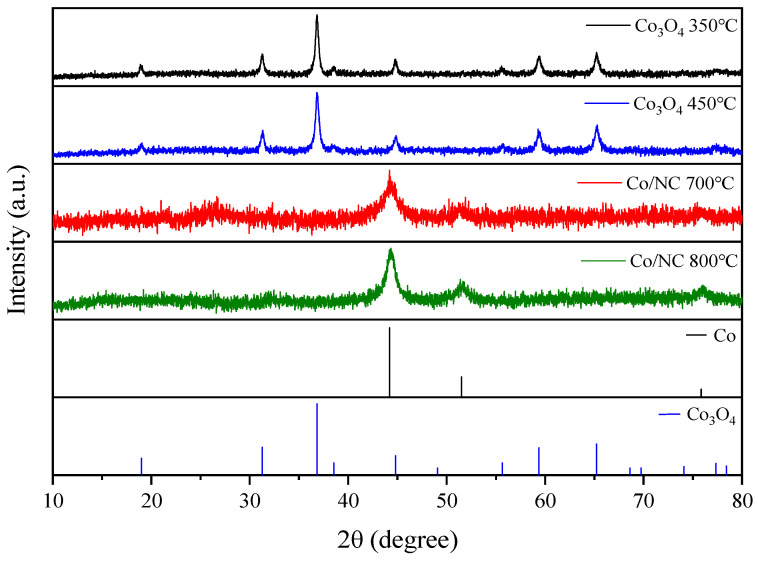
XRD patterns of Co/NC-700, Co/NC-800, Co_3_O_4_-450, and Co_3_O_4_-350.

The crystalline structures of Co/NC-700, Co/NC-800, Co_3_O_4_-450, and Co_3_O_4_-350 were analyzed using powder XRD (Figure 3), and the absence of peaks corresponding to ZIF-67 indicates the successful conversion into their respective derivatives. Co/NC-700 and Co/NC-800, prepared by calcination under a nitrogen atmosphere, displayed distinct peaks at 44.25°, 51.48°, and 75.8°, corresponding to the (002), (101), and (110) planes of cobalt (PDF#15-0806), confirming the formation of metallic cobalt with a well-ordered crystalline structure. The high-intensity peaks observed suggest a significant degree of crystallinity in the cobalt phase, which can enhance the material’s conductivity and improve its performance in electrochemical applications. On the other hand, Co_3_O_4_-450 and Co_3_O_4_-350, synthesized by oxidation in air, exhibited peaks at 18.99°, 31.27°, 36.79°, 38.4°, 44.79°, 55.52°, 59.25°, and 65.16°, which are characteristic of the cubic Co_3_O_4_ phase (PDF#74-2120). The presence of these peaks indicates the formation of a Co_3_O_4_ spinel structure, known for its high oxidation state and strong interaction with oxygen species. The higher oxygen content in Co_3_O_4_ samples, as indicated by XPS analysis, further supports the formation of this oxide phase. The difference in the crystalline phases between the Co/NC and Co_3_O_4_ samples highlights the influence of the calcination atmosphere on the final material structure. Nitrogen calcination favors the formation of metallic cobalt, which offers superior electrical conductivity, while air oxidation leads to cobalt oxide, which may exhibit different electrochemical properties. This structural variation is critical for tailoring the materials for specific applications, where the nature of the active phase plays a crucial role in determining performance.

**Figure 4:**
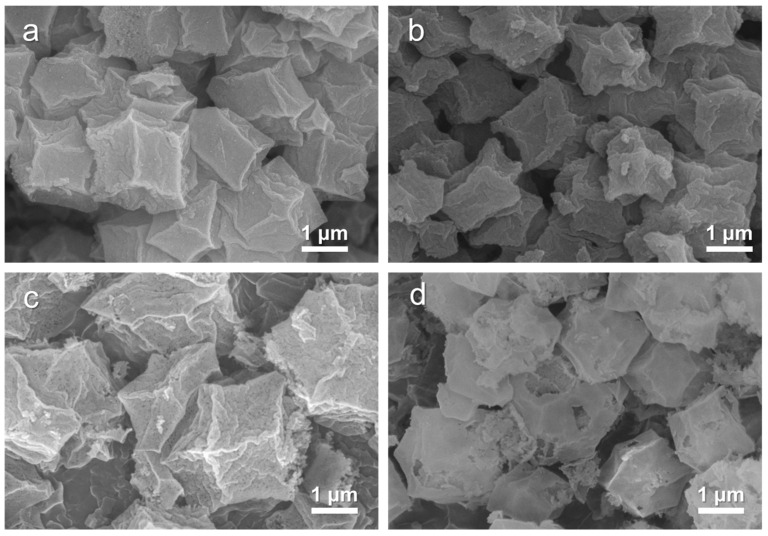
SEM images of (**a**) Co/NC-700, (**b**) Co/NC-800, (**c**) Co_3_O_4_-450, and (**d**) Co_3_O_4_-350.

The morphologies of Co/NC-700, Co/NC-800, Co_3_O_4_-450, and Co_3_O_4_-350 were examined using SEM at both low and high magnifications to assess their surface microstructures. As shown in Figure 4a, Co/NC-700 retains its rhombic dodecahedron shape, with its surface remaining intact after hydrofluoric acid etching and a particle size of approximately 800–1000 nm. In Figure 4b, Co/NC-800 exhibits slight deformation, likely due to the high carbonization temperature. Co_3_O_4_-450 (Figure 4c) also retains a dodecahedral structure, though some surface damage is observed, with a size of around 700–900 nm. Co_3_O_4_-350 (Figure 4d) presents a larger rhombic dodecahedron structure with dimensions of about 1.2–1.3 µm. EDS mapping (Figures S1–S4) indicates a uniform distribution of C, O, and Co across all samples.

**Figure 5:**
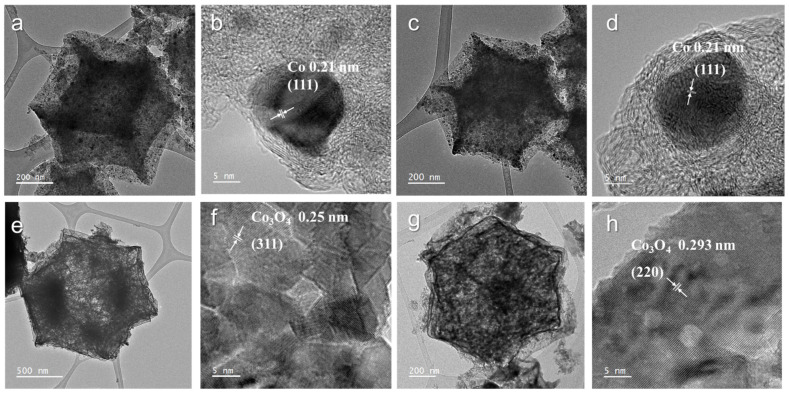
TEM and HRTEM images of (**a**,**b**) Co/NC-700, (**c**,**d**) Co/NC-800, (**e**,**f**) Co_3_O_4_-450, and (**g**,**h**) Co_3_O_4_-350.

**Figure 6:**
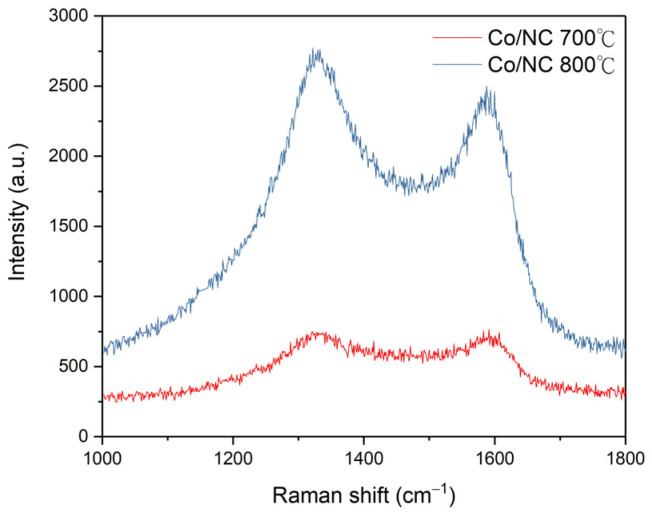
Raman spectrum of Co/NC-700 and Co/NC-800.

The crystalline structures of Co/NC-700, Co/NC-800, Co_3_O_4_-450, and Co_3_O_4_-350 (Figure 5) were further analyzed using TEM and HR-TEM. For Co/NC-700 and Co/NC-800, a lattice spacing of 0.20 nm corresponds to the (111) plane of the Co crystal phase. These results indicate that calcination in a nitrogen atmosphere promotes the formation of cobalt nanoparticles, aligning with the XRD analysis. In contrast, Co_3_O_4_-450 and Co_3_O_4_-350 exhibit lattice spacings of 0.25 nm and 0.293 nm, which correspond to the d-spacings of the (311) and (220) planes of Co_3_O_4_, respectively. Raman spectroscopy was employed to assess the graphitization degree of the samples. Co_3_O_4_-350 and Co_3_O_4_-450, being oxidation products, do not show D and G peaks at around 1300 cm^−1^ and 1600 cm^−1^, indicating a low degree of graphitization. In contrast, the Co/NC-700 and Co/NC-800 samples exhibit I_D_/I_G_ ratios of 1.29 and 1.82, respectively (Figure 6). The higher ratio for Co/NC-800 suggests that at calcination temperatures of 700–800 °C, the organic ligands and metal nodes were largely removed, leading to increased structural defects, which is reflected in the higher I_D_/I_G_ ratio for Co/NC-800.

**Figure 7:**
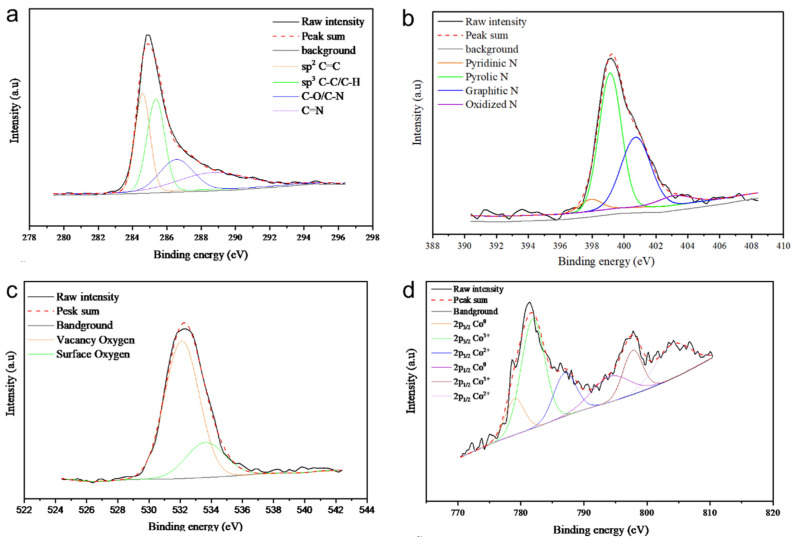
High-resolution XPS spectra of Co/NC-700, showing (**a**) C 1s, (**b**) N 1s, (**c**) O 1s, and (**d**) Co 2p.

**Figure 8:**
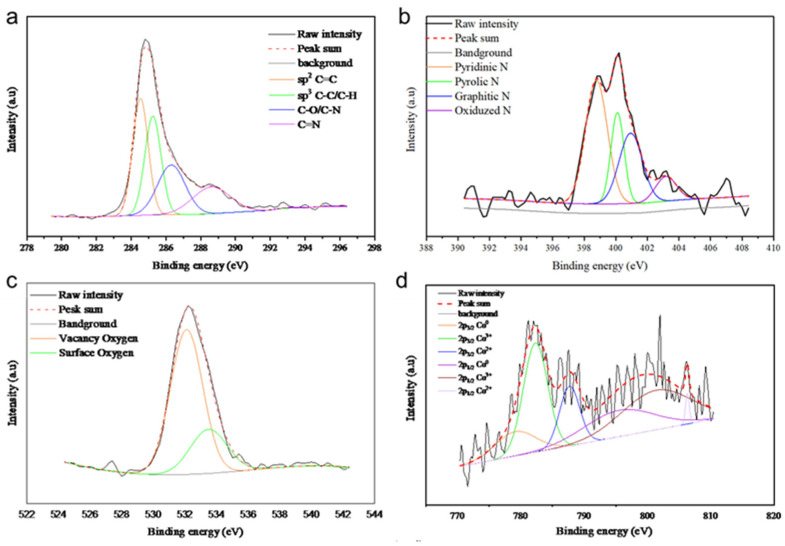
High-resolution XPS spectra of Co/NC-800, showing (**a**) C 1s, (**b**) N 1s, (**c**) O 1s, and (**d**) Co 2p.

**Figure 9:**
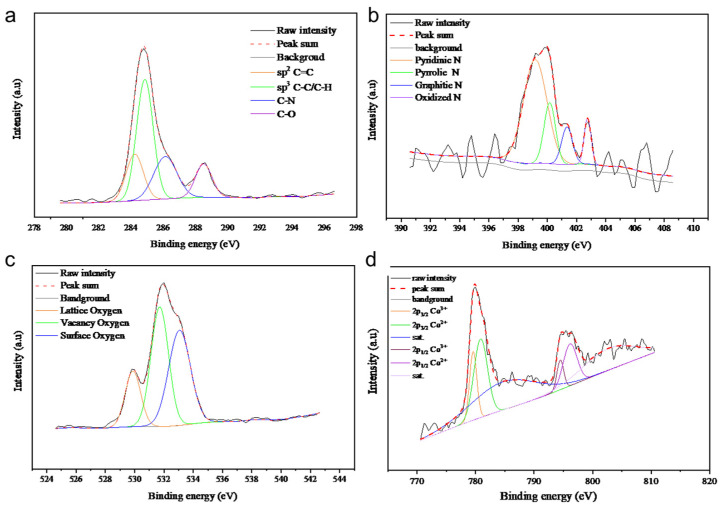
High-resolution XPS spectra of Co_3_O_4_-450, showing (**a**) C 1s, (**b**) N 1s, (**c**) O 1s, and (**d**) Co 2p.

**Figure 10:**
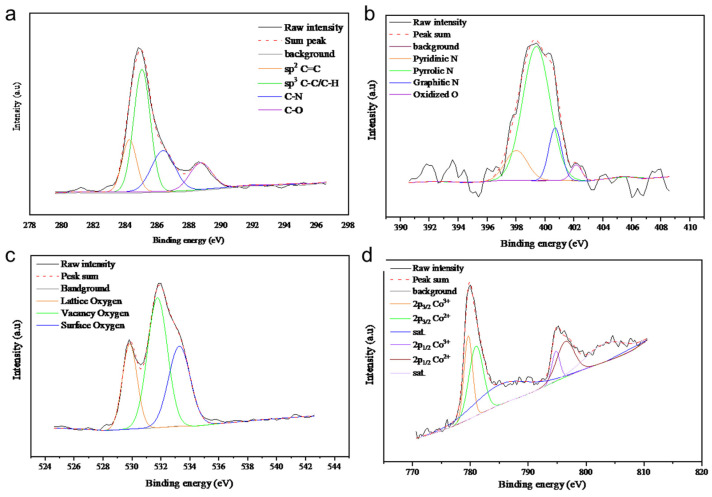
High-resolution XPS spectra of Co_3_O_4_-350, showing (**a**) C 1s, (**b**) N 1s, (**c**) O 1s, and (**d**) Co 2p.

The XPS analysis of all samples is presented in Figure 7, Figure 8, Figure 9 and Figure 10, with the elemental compositions summarized in Table 1. The C 1s XPS spectra were deconvoluted into four components: sp^2^ C=C (284.6 eV), sp^3^ C-C (285.2 eV), C-O/C-N (286.9 eV), and C=N (289.0 eV). The N 1s spectra were fitted with four peaks corresponding to pyridinic N (398.5 eV), pyrrolic N (399.0 eV), quaternary N (400.2 eV), and graphitic N (402.1 eV), originating from the ZIF-67 precursor. Co/NC-700 and Co/NC-800 samples showed higher nitrogen contents compared to the other samples, indicating that nitrogen was retained during the calcination process under an N_2_ atmosphere. The O 1s spectra revealed three distinct peaks, attributed to lattice oxygen, vacancy oxygen, and surface oxygen [23]. Co/NC-700 and Co/NC-800 exhibited higher carbon content, while Co_3_O_4_-450 and Co_3_O_4_-350 showed higher oxygen content, indicating a successful oxidation process that transformed the ZIF-67 precursor into cobalt oxide, in line with the XRD results. Moreover, Co/NC-700 and Co/NC-800 not only have a higher carbon ratio but also a significantly greater sp^2^ C=C content compared to Co_3_O_4_-450 and Co_3_O_4_-350, which could enhance their electrical conductivity. The Co 2p spectra showed two shakeup satellites (sat.) and peaks at 779.6 eV and 794.5 eV, corresponding to the Co^3+^ 2p_3/2_ and Co 2p_1/2_ orbitals, respectively. Peaks at 780.9 eV and 796.1 eV were attributed to Co^2+^.

VRFBs were constructed using electrodes coated with Co/NC-700, Co/NC-800, Co_3_O_4_-350, and Co_3_O_4_-450 on GF as the positive electrode, while uncoated GF was employed as the negative electrode. These configurations were denoted as Co/NC-700//GF, Co/NC-800//GF, Co_3_O_4_-350//GF, and Co_3_O_4_-450//GF, respectively, to distinguish the different electrode materials and preparation methods. For comparative purposes, a control VRFB was also assembled using uncoated GF for both the positive and negative electrodes (denoted as GF//GF) to evaluate how the presence of active material coatings affects the overall performance of the cells. This experimental setup aimed to investigate the influence of electrode material on critical parameters such as charge transfer resistance, voltage efficiency (VE), coulombic efficiency (CE), and energy efficiency (EE).

**Figure 11:**
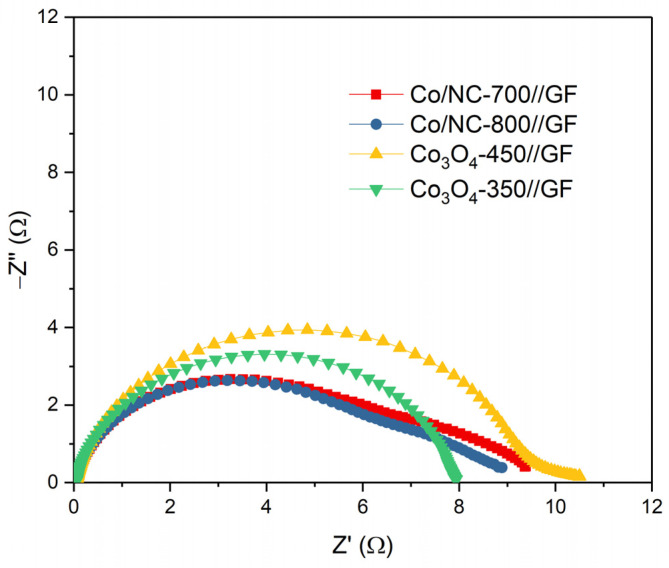
The Nyquist plots of Co/NC-700//GF, Co/NC-800//GF, Co_3_O_4_-350//GF, and Co_3_O_4_-450//GF VRFB.

Figure 11 shows the Nyquist plots for all samples, which were measured at 0 V in an electrolyte solution of 1.6 M VOSO_4_ in 3 M H_2_SO_4_ over a broad frequency range from 10^−3^ Hz to 10⁶ Hz. The initial intersection of the semicircular arc with the real axis corresponds to the solution’s ohmic resistance (R_s_), which reflects the resistance of the electrolyte and the contacts within the cell. Importantly, R_s_ remained consistent across all electrodes, suggesting that the electrolyte composition and electrode contact in the VRFBs were uniform. The high-frequency semicircular arc represents the charge transfer process at the electrode/electrolyte interface, with the charge transfer resistance (R_ct_) indicating the ease with which electrons can move across this interface. The R_s_ and R_ct_ values for each electrode are provided in Table 2, revealing significant differences in R_ct_ after coating the electrodes with active materials. Specifically, the control GF//GF configuration exhibited a relatively high R_ct_ of 44.80 Ω, while the electrodes coated with Co/NC-700 and Co/NC-800 showed dramatically lower R_ct_ values of 6.41 Ω and 6.42 Ω, respectively. The R_ct_ values of Co_3_O_4_-450//GF and Co_3_O_4_-350//GF were 9.39 Ω and 12.00 Ω. The reduction in R_ct_ is likely attributed to the higher calcination temperatures used in the preparation of Co/NC-700 and Co/NC-800, which enhance carbon content and promote a higher degree of graphitization, resulting in improved electrical conductivity, as corroborated by the XPS analysis.

**Figure 12:**
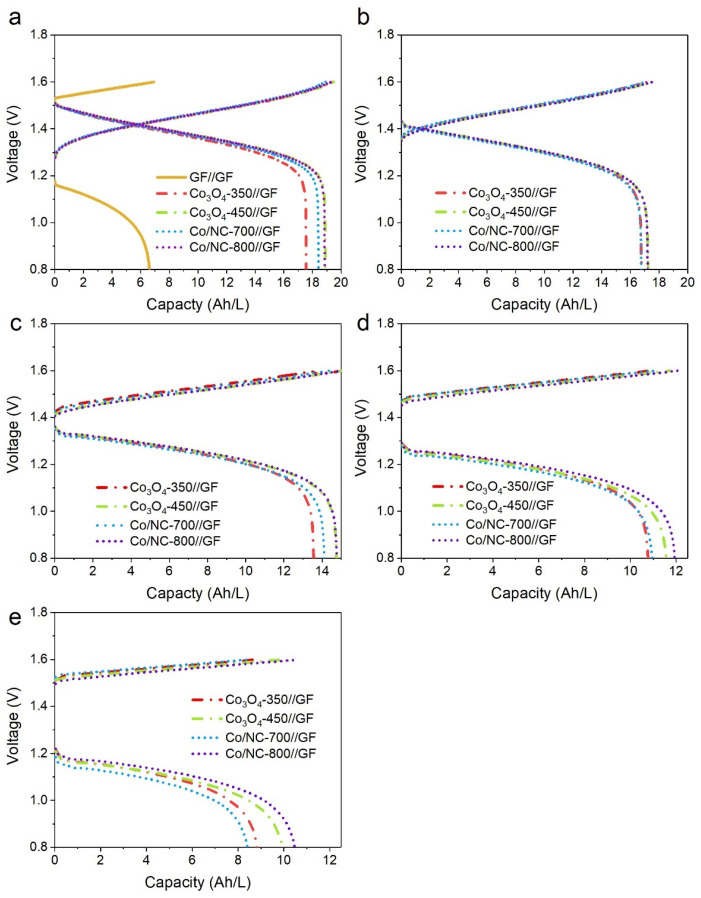
Charge–discharge curves of GF//GF, Co/NC-700//GF, Co/NC-800//GF, Co_3_O_4_-350//GF, and Co_3_O_4_-450//GF VRFB at the current densities of (**a**) 50, (**b**) 100, (**c**) 150, (**d**) 200, and (**e**) 250 mA cm^−2^.

**Figure 13:**
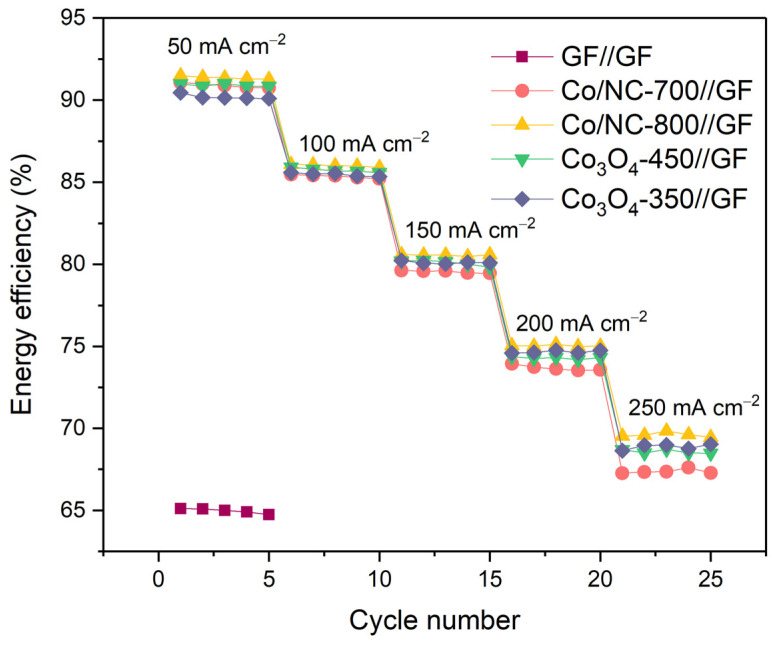
Energy efficiency (EE) of VRFB assembled with GF//GF, Co/NC-700//GF, Co/NC-800//GF, Co_3_O_4_-350//GF, and Co_3_O_4_-450//GF.

To evaluate the full-cell performance, charge–discharge tests were conducted at various current densities (50, 100, 150, 200, and 250 mA cm^−2^) (Figure 12). The operating temperature was 30 ± 5 °C, and the VRFB was charged and discharged to the cutoff voltages of 1.6 V and 0.8 V. These tests provided insights into the electrodes’ performance under different operating conditions. The results were summarized in Figure 13 and Table 3, where the average values of VE, CE, and EE were measured over five cycles. VE is directly related to the internal resistance of the electrodes, while CE is influenced by the efficiency of ion transport across the membrane, which ultimately impacts the EE of the VRFB system. All coated electrodes (Co/NC-700//GF, Co/NC-800//GF, Co_3_O_4_-350//GF, and Co_3_O_4_-450//GF) demonstrated excellent CE values of approximately 98% across all current densities, indicating efficient ion transport through the membrane. In contrast, the control GF//GF configuration exhibited a slightly lower CE of 96.04%, reflecting its comparatively poorer ion permeability. At the lowest current density of 50 mA cm^−2^, the control GF//GF cell displayed VE and EE values of 67.80% and 64.97%, respectively, indicating relatively high internal resistance. As the current density increased, the GF//GF cell failed to maintain stable charge–discharge cycles, likely due to its high resistance and lack of active materials to facilitate the redox reactions. In contrast, the active material-coated electrodes exhibited significantly better performance. At 50 mA cm^−2^, the EE values for Co/NC-700//GF, Co/NC-800//GF, Co_3_O_4_-350//GF, and Co_3_O_4_-450//GF were 90.89%, 91.37%, 90.91%, and 90.19%, respectively. Their discharge capacities were 18.41, 18.45, 18.90, and 18.50 Ah L^−1^, respectively, demonstrating comparable performance across all active material-coated electrodes. Notably, the uncoated GF//GF electrode achieved a significantly lower discharge capacity of only 6.62 Ah L^−1^, highlighting the substantial impact of the active material coatings, which resulted in a performance improvement of approximately 178%. At the highest current density of 250 mA cm^−2^, the VE values for Co/NC-700//GF, Co/NC-800//GF, Co_3_O_4_-350//GF, and Co_3_O_4_-450//GF were 68.00%, 70.38%, 69.39%, and 69.66%, respectively, while their corresponding EE values were 67.37%, 69.61%, 68.58%, and 68.88%. Among these, Co/NC-800//GF stood out as the best-performing electrode, delivering the highest discharge capacity of 10.49 Ah L^−1^, showcasing its superior ability to sustain high power output. This enhanced performance at higher current densities can be attributed to the increased graphitization degree and optimized conductivity of the nitrogen-doped carbon structure in Co/NC-800, which allows for more efficient charge transfer and redox reaction kinetics. The observed results clearly demonstrate that the incorporation of active materials, particularly Co/NC-800, significantly improves the electrochemical performance of the VRFB system. These findings highlight the importance of optimizing electrode materials for enhanced energy efficiency, lower internal resistance, and greater discharge capacity, especially under varying operating conditions.

**Figure 14:**
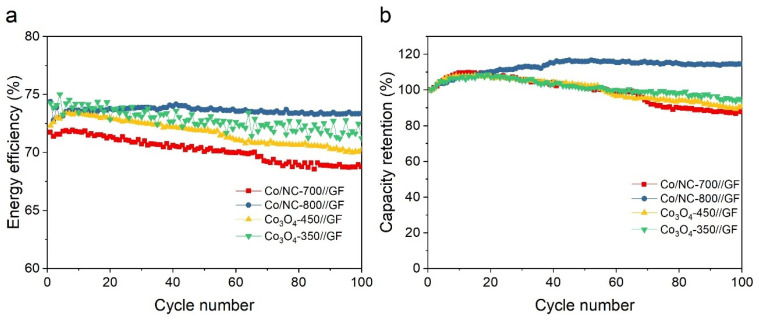
(**a**) EE and (**b**) capacity retention of all VRFBs during 100 cycles at 200 mA cm^−2^.

To further evaluate the stability of the electrodes, the Co/NC-700//GF, Co/NC-800//GF, Co_3_O_4_-350//GF, and Co_3_O_4_-450//GF electrodes were subjected to 100 charge–discharge cycles at 200 mA cm^−2^, assessing their discharge capacity decay. Figure 14a shows the EE values for these electrodes, which were 68.75%, 73.37%, 70.20%, and 71.23%, respectively, and remained stable over the entire cycling period. Co/NC-800//GF maintained the highest EE after 100 cycles. The fluctuation of the EE value for Co_3_O_4_-350//GF is due to its higher resistance. The charge transfer resistance affects electrochemical reactions, leading to instability in battery performance over long periods of operation. Figure 14b shows the capacity retention, which were 87.86%, 114.60%, 91.10%, and 93.96%, respectively. All VRFBs experienced an initial increase in discharge capacity during the first 20 cycles, followed by different trends. Co/NC-700//GF, Co_3_O_4_-350//GF, and Co_3_O_4_-450//GF exhibited gradual capacity decay, whereas Co/NC-800//GF continued to improve, eventually achieving 114.60% of its initial capacity. This improvement is likely due to the activation of the active material, enhancing the redox reactions at the electrode surface. As a result, Co/NC-800 emerged as the most promising electrode material for VRFBs, demonstrating outstanding electrochemical performance, long-term stability, and high energy efficiency. The differences in electrochemical performance among the electrodes—Co/NC-700, Co/NC-800, Co_3_O_4_-450, and Co_3_O_4_-350—can be attributed to their distinct material properties. A key factor influencing the electrochemical behavior is the formation of different crystalline phases. Co/NC-700 and Co/NC-800, calcined in a nitrogen atmosphere, contain metallic cobalt nanoparticles with well-ordered crystalline structures, higher carbon content, and a higher degree of graphitization. The superior graphitization in Co/NC-800, reflected by its higher ID/IG ratio from Raman spectroscopy, further enhances electron mobility, particularly at higher current densities. This structural advantage explains Co/NC-800′s superior performance, especially during high-power operation. In contrast, Co_3_O_4_-450 and Co_3_O_4_-350, calcined in an oxidative air atmosphere, form a Co_3_O_4_ spinel structure, which has lower electrical conductivity compared to metallic cobalt. While cobalt oxide can participate in redox reactions, its reduced conductivity hinders efficient charge transfer, leading to higher R_ct_ values. Consequently, these electrodes show slightly inferior electrochemical performance compared to Co/NC-700 and Co/NC-800. These differences in electrochemical behavior underscore the importance of optimizing material synthesis to achieve the desired phase, structure, and composition for specific electrochemical applications like VRFBs. Additionally, this study highlights the potential of ZIF-67-derived materials to advance the development of efficient and durable VRFB systems for large-scale energy storage applications.

## 4. Conclusions

In summary, we successfully synthesized Co/NC-700, Co/NC-800, Co_3_O_4_-350, and Co_3_O_4_-450 electrode materials using ZIF-67 as a template for VRFBs. These materials were evaluated for their electrochemical performance, including wettability, structural characteristics, and energy efficiency in a full-cell setup. The calcination processes in both nitrogen and air atmospheres allowed for the creation of cobalt-based derivatives that demonstrated enhanced electrocatalytic activity and improved charge–discharge characteristics. The electrochemical performance of the VRFBs assembled with these coated electrodes demonstrated significant improvements in comparison to the uncoated GF control. Notably, the charge transfer resistance (R_ct_) of the Co/NC-700//GF and Co/NC-800//GF electrodes was significantly reduced to 6.41 Ω and 6.42 Ω, respectively, compared to 44.80 Ω for the uncoated GF//GF electrode. This reduction in R_ct_, along with the increased conductivity and enhanced graphitization from high-temperature calcination, contributed to the superior performance of these electrodes. In charge–discharge tests, the Co/NC-800//GF demonstrated the best overall performance. At a current density of 50 mA cm^−2^, the Co/NC-800//GF configuration achieved an energy efficiency (EE) of 91.37%, voltage efficiency (VE) of 93.04%, and coulombic efficiency (CE) of 98.08%. Even at a higher current density of 250 mA cm^−2^, the Co/NC-800//GF maintained high performance with EE, VE, and CE values of 69.61%, 70.38%, and 98.07%, respectively, highlighting its suitability for high-power applications. In contrast, the control GF//GF cell showed significantly lower performance across all current densities, with an EE of only 64.97% and discharge capacity of 6.62 Ah L^−1^ at 50 mA cm^−2^. The long-term cycling stability of the electrodes was also assessed. After 100 cycles at 200 mA cm^−2^, Co/NC-800 maintained an energy efficiency of 73.37% and achieved a capacity retention of 114.60%, further emphasizing its durability and effectiveness in VRFB applications. This remarkable capacity retention suggests that the electrode experienced a favorable activation process during cycling, enhancing its overall performance.

## Figures and Tables

**Table 1:** The atomic percentages of all the samples.

Sample	C (at.%)	O (at.%)	Co (at.%)	N (at.%)
Co/NC-700	74.1	13.0	4.2	8.7
Co/NC-800	68.3	21.2	2.1	8.4
Co_3_O_4_-450	58.2	30.9	6.5	4.5
Co_3_O_4_-350	64.5	28.9	4.1	2.5

**Table 2:** The R_s_ and R_ct_ values of GF//GF, Co/NC-700//GF, Co/NC-800//GF, Co_3_O_4_-450//GF, and Co_3_O_4_-350//GF.

Sample	R_s_	R_ct_
pristine graphite felt	0.03	44.80
Co/NC-700	0.04	6.41
Co/NC-800	0.03	6.42
Co_3_O_4_-450	0.05	9.39
Co_3_O_4_-350	0.05	12.00

**Table 3:** Electrochemical data obtained from the charge–discharge tests.

Positive Electrode	Current Density (mA cm^−2^)	Average VE (%)	Average CE (%)	Average EE (%)	Discharge Capacity (Ah/L)
GF//GF	50	67.80	96.04	64.97	6.62
Co/NC-700//GF	50	92.69	98.06	90.89	18.41
	100	86.69	98.46	85.35	16.77
	150	80.66	98.62	79.54	14.11
	200	74.53	98.85	73.68	10.97
	250	68.00	99.06	67.37	8.44
Co/NC-800//GF	50	92.97	98.28	91.37	18.45
	100	87.28	98.55	86.01	17.21
	150	81.65	98.66	80.55	14.77
	200	76.00	98.74	75.04	11.94
	250	70.38	98.90	69.61	10.49
Co_3_O_4_-450//GF	50	93.49	97.49	90.91	18.90
	100	87.28	98.22	85.73	17.25
	150	81.36	98.42	80.08	14.76
	200	75.32	98.64	74.29	11.57
	250	69.39	98.83	68.58	9.97
Co_3_O_4_-350//GF	50	92.89	97.09	90.19	18.50
	100	87.19	98.02	85.46	16.78
	150	81.36	98.46	80.11	13.58
	200	75.71	98.62	74.67	10.79
	250	69.66	98.88	68.88	8.82

## Data Availability

Data are contained within the article and Supplementary Materials.

## Supplementary Materials

The following supporting information can be downloaded at: https://www.mdpi.com/article/10.3390/molecules29215061/s1, Figure S1. EDS mapping of Co/NC-700. Figure S2. EDS mapping of Co/NC-800. Figure S3. EDS mapping of pfCo_3_O_4_-450. Figure S4. EDS mapping of Co_3_O_4_-35.
