# Supplementary material for: Enhancing Vanadium Redox Flow Battery Performance with ZIF-67-Derived Cobalt-Based Electrode Materials
Source: Molecules. 2024 Oct 26;29(21):5061. doi: 10.3390/molecules29215061 (PMC11547396; doi:10.3390/molecules29215061)
Supplement: Supplementary file 1 [file molecules-29-05061-s001.zip › molecules-3253985-supplementary.pdf]

Enhancing Vanadium Redox Flow Battery Performance with ZIF-67-Derived Cobalt-Based Electrode Materials

Christine Young [1,*], Zhen-Qi Liao [1], Dong-Rong Li [1], Pei-Ling Li [1], Chen-Yang Wang [1], Shu-Mei Ho [2] and Chi-Chang Chen [2,*]

1 Department of Chemical and Materials Engineering, National Yunlin University of Science and Technology, Yunlin 640301, Taiwan.

2 Green Energy and Environment Research Laboratories, Industrial Technology Research Institute, Tainan 711010, Taiwan.

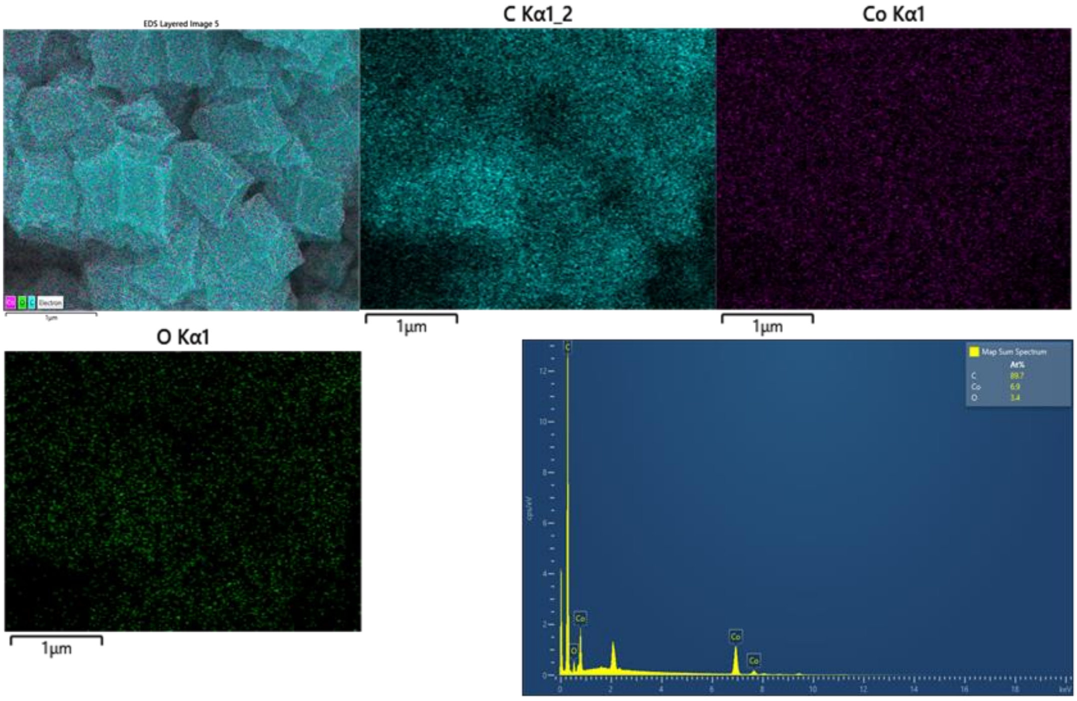

Figure S1. EDS mapping of Co/NC-700

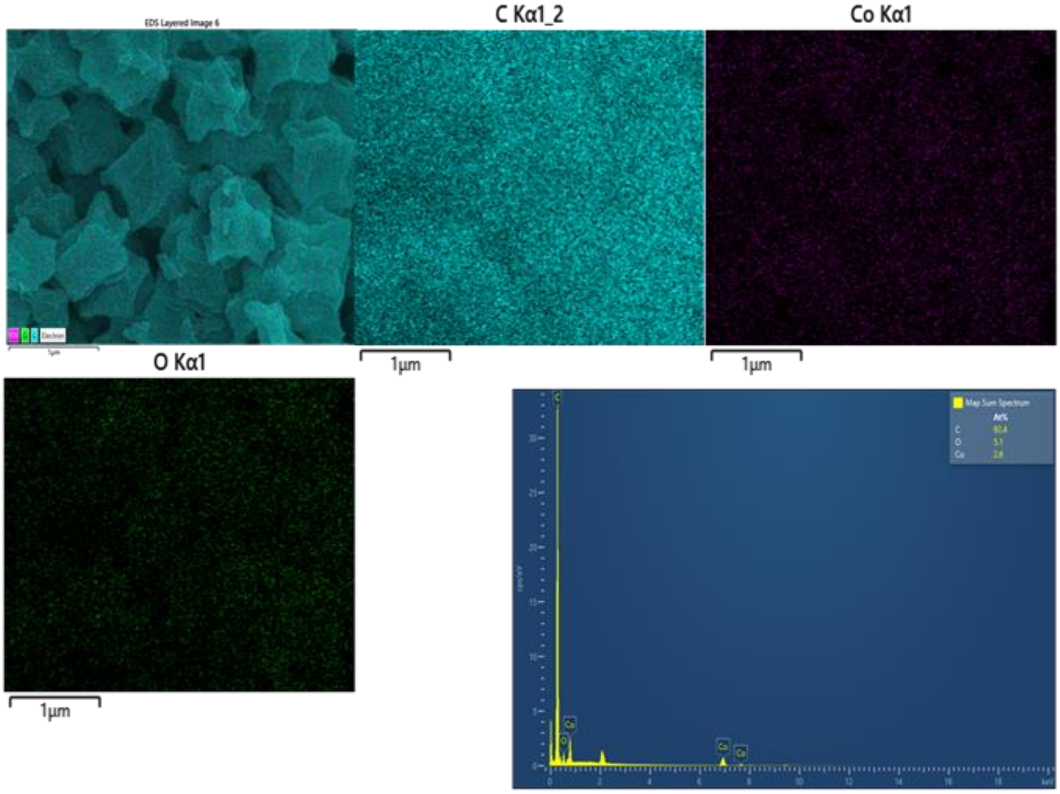

Figure S2. EDS mapping of Co/NC-800

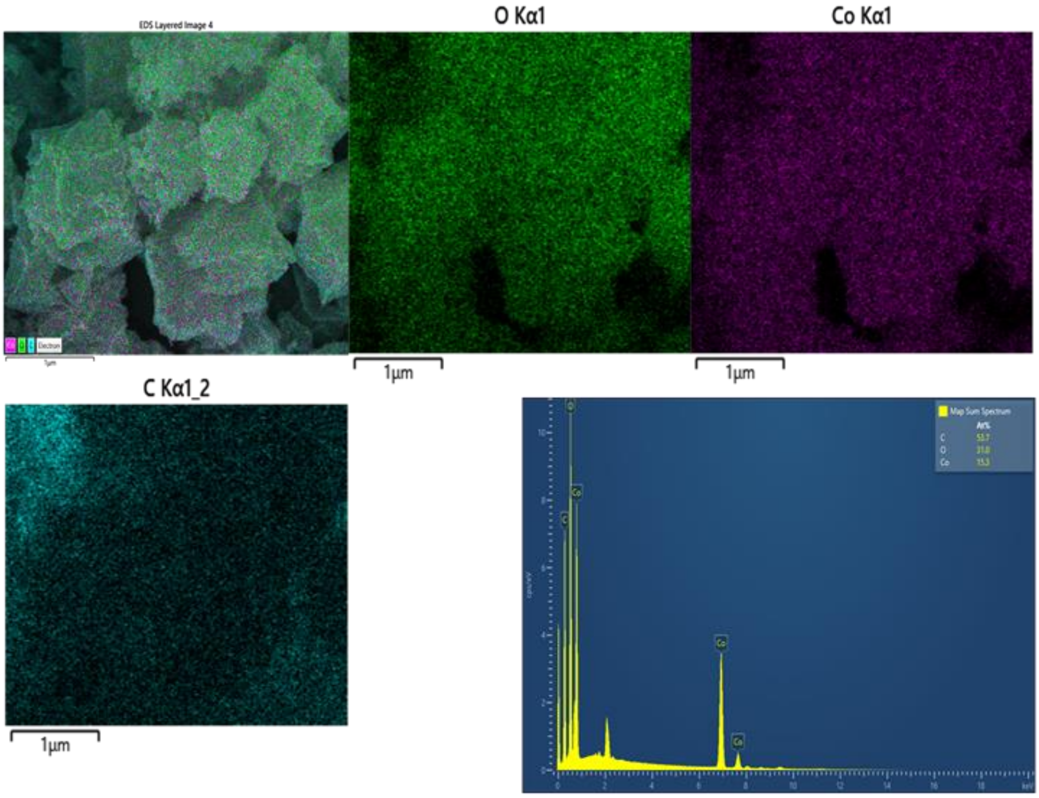

Figure S3. EDS mapping of pfCo_3O_4-450

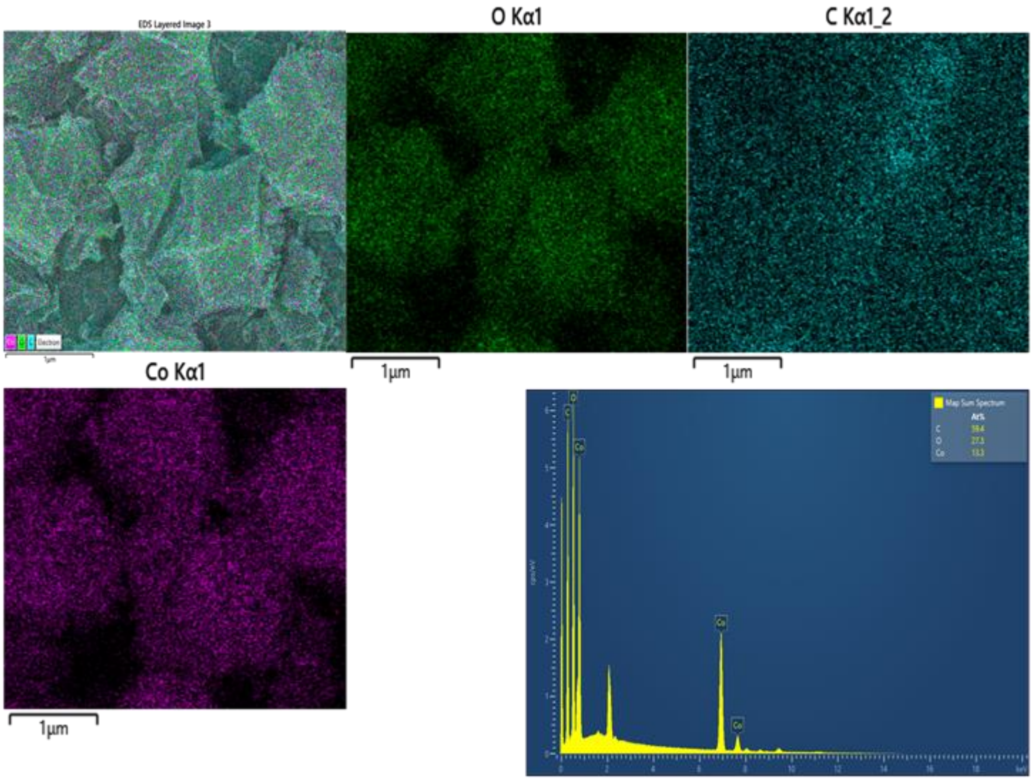

Figure S4. EDS mapping of Co_3O_4-35
